# Biomechanical study of the abductor pollicis longus & extensor pollicis brevis after release and reconstruction of the first dorsal compartment of the wrist^☆^

**DOI:** 10.1016/j.amsu.2021.102863

**Published:** 2021-09-15

**Authors:** Amit Gupta, Fateme Mirzaee, Amir Farahanchi Baradaran, Mohammad Amin Aslani, Hamidreza Aslani

**Affiliations:** a Director of Louisville Arm and Hand Surgery, USA; b MSc of Orthosis & Prosthesis, University of Social Welfare and Rehabilitation Sciences, Knee and Sport Medicine Research Center, Milad Hospital, Tehran, Iran; c Shahid Beheshti University of Medical Sciences, Knee and Sport Medicine Research Center, Milad Hospital, Tehran, Iran; d Medical Student of Tehran University of Medical Sciences, Knee and Sport Medicine Research Center, Milad Hospital, Tehran, Iran

**Keywords:** Abductor pollicis longus, Biomechanics, First dorsal compartment, Release

## Abstract

**Background:**

The purpose of this study was to determine the biomechanical changes after first dorsal compartment release and after Z-plasty of the first dorsal compartment with respect to the excursion and displacement of the abductor pollicis longus and extensor pollicis brevis tendons.

**Materials and methods:**

Six fresh frozen cadaveric hand and forearms were obtained and the tendons of the abductor were exposed and separated from surrounding tissues through a palmar incision and tenotomized at the musculotendinous junction. The excursion and displacement of the abductor pollicis brevis and flexor pollicis brevis tendons were measured for all six cadaveric hands.

**Results:**

Increases in tendon excursion were observed in both the abductor pollicis longus (29%) and extensor pollicis brevis (30%) while these observations after Z-plasty were 0.04% for abductor pollicis longus and 0.07% for extensor pollicis brevis. With the use of the modified Elftman curve for the Lengths, tension relationship and Brand's data for resting fiber length we found that 1 cm increase of The excursion of the APL and EPB will decrease tension %65 and %80 respectively. There was also a 12.2-mm displacement of the tendons after release and 4.8 mm displacement after z-plasty of the compartment.

**Conclusion:**

It seems that Procedures such as enlargement plasty of Kapandji or Z-plasty will increase the size of the compartment but will not change the biomechanical behaviors of the tendons significantly.

## Highlights

• One of the common causes of wrist and hand pain or disability is the first extensor compartment entrapment.
• Surgical approaches for de Quervain tenosynovitis may lead to volar subluxation or excursion of the APL and EPB tendons.
• The purpose of this study was to determine the biomechanical changes after first dorsal compartment release.

## 1 Introduction

The first extensor wrist compartment lies over the radius styloid process and encompasses the abductor pollicis longus (APL) and extensor pollicis brevis (EPB) tendons [1]. One of the common causes of wrist and hand pain or disability is the first extensor compartment entrapment. For the first time Fritz de Quervain describes the tenosynovitis APL and EPB tendons [2,3]. Despite great interest in this case, its pathology and leading cause remain unclear [4,5]. By considering histopathologic investigations, de Quervain's disease is the consequence of tendon sheath thickening due to the myxoid degeneration by mucopolysaccharide accumulation [5] and inflammation does not play a significant role in its occurrence. These changes could consider as de Quervain's disease characteristics. Up to now several studies have demonstrated a relationship between de Quervain's disease, pregnancy, and lactation [6]. Several approaches have been recommended in order to treat this disease [2,7]. Commonly, the first step in de Quervain's disease management is conservative approach, but surgery should be considered if nonoperative treatments fail for 4–6 months [6,7]. Most surgeons believe that abnormal compartmanization can lead to the process and maybe explain the undesired results of nonoperative management in certain patient populations [2,7,8]. In this condition, operative release of the first extensor compartment perhaps inevitable. The most frequent surgical approaches for de Quervain tenosynovitis includes releasing the first extensor wrist compartment, excision of the extensor retinaculum Kapandji enlargementplasty of the first dorsal compartment [9], W-plasty and Z-plasty of the first dorsal compartment. These methods can bring about satisfactory outcomes but sometimes may lead to volar subluxation or excursion of the APL and EPB tendons [7,10,11]. Other surgical complications of first dorsal compartment release are uncommon and generally because of injury to the superficial branch of the radial nerve or because of imperfect release or re-adhesion of the extensor retinaculum [7,9,12]. The purpose of this study was to determine the biomechanical changes after first dorsal compartment release and after Z-plasty of the first dorsal compartment with respect to the excursion and displacement of the APL and EPB tendons.

## 2 Materials and methods

**Fig. 1:**
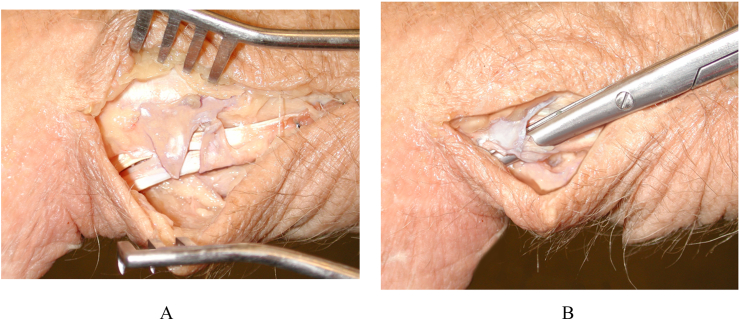
A. After release of tendons of the adductor pollicis and flexor pollicis brevis B. After Reconstruction.

**Fig. 2:**
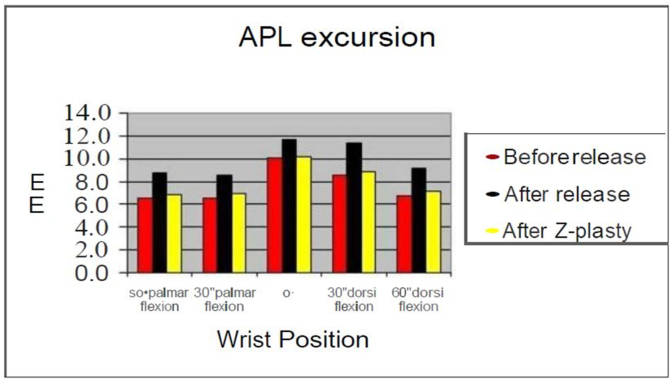
Abductor Policies Longus excursion before release, after release and after Z-plasty of the first dorsal compartment.

**Fig. 3:**
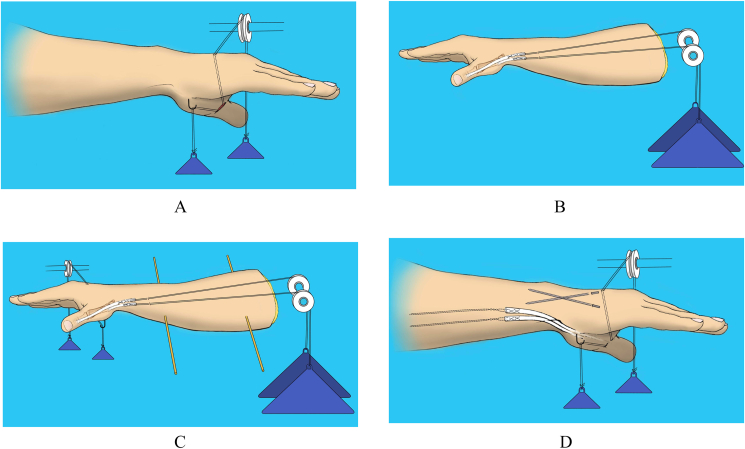
Measurement methods. A. Traction line applied with 2-0 prolene with 100 gr. B. 700 gr. traction with 0 prolene. C. Forearm secured with two Steinmann pins through the radius. D. Wrist pinned in 5 positions.

Six fresh frozen cadaveric hand and forearms were obtained and underwent X-ray investigation in order to rule out any bony pathology. The tendons of the adductor pollicis and flexor pollicis brevis were exposed and separated from surrounding tissues through a palmar incision and tenatomised at the musculotendinous junction and in-line traction was applied using 2-0 prolene and 100g (Fig. 1). The APL and EPB tendons were explored through a second incision on dorsum of the forearm and tenatomised at the musculotendinous junction and in-line traction was applied using 0 prolene and 700g. Greater amount of traction were found to result in lengthening of the tendons'suture unit especially in EPB as demonstrated by the tendon not returning to its previous position in subsequent trials. Each forearm was secured horizontally palm down with two heavy steinmann like pins through proximal and distal ends of the radius and ulna and mounted in a loading apparatus (Fig. 2). The wrist was fixed with two steinmann pins in 60° palmar flexion, 30° palmar flexion, 0° flexion and extension, 30° dorsal flexion and 60° dorsal flexion. The interphalangeal joint was fixed and the thumb was moved through a range of motion (R.O.M) for abduction at carpometacarpal joint and extension of the metacrpophalangeal joint. The metacrpophalangeal and the carpometacarpal joint was fixed for each tendon respectively and defined as start point. The minimum significant amount of APL and EPB tendon excursions that was measured individually using a caliper was 0.1 mm. A marker on the tendons and a fixed point on the dorsum of the forearm were used as references. Then in each position, the wrist was fixed in radial and ulnar deviation and displacement of the APL and EPB tendons was measured (Fig. 3). This procedure was repeated after release of the first dorsal compartment and after suturing of the limbs of Z-plasty of the compartment and all measurement was repeated and recorded. Student's *t-*test was used to evaluate the outcome of variables. The study has been reported in line with the PROCESS consensus preferred reporting of case series in surgery (PROCESS) guidelines [13].

## 3 Results

**Fig. 4:**
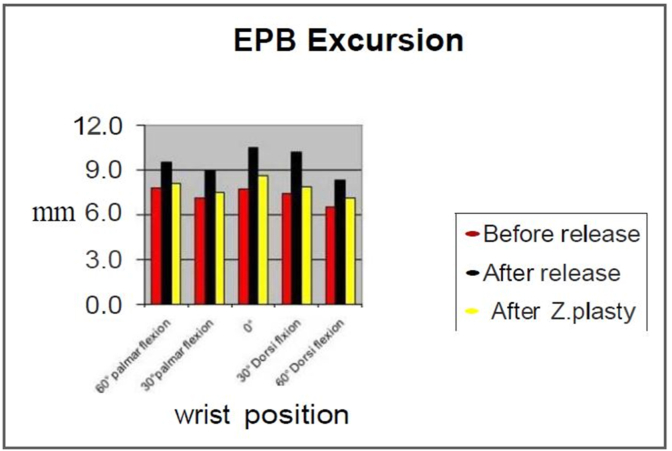
Extensor Pollicis Brevis excursion in various Degree of wrist position before release after release and after Z-plasty of the first dorsal compartment.

**Fig. 5:**
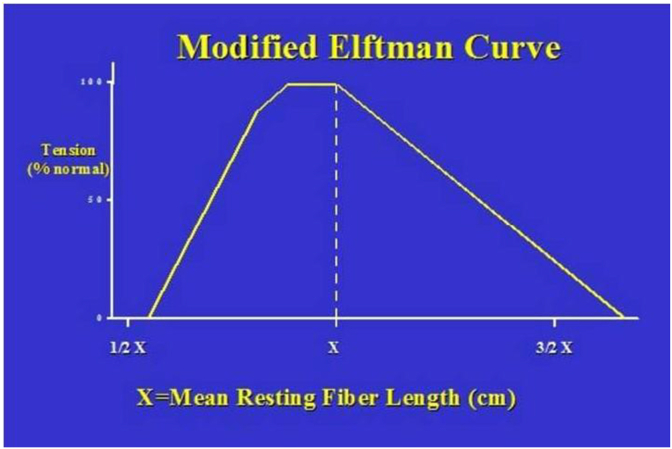
Demonstrated modified Elftman curve.

The excursion and displacement of the APL and EPB tendons were measured for all six cadaveric hands. A total of 390 observations were made regarding the amount of APL and EPB tendon displacement and excursion. Ninety of these observations involved APL excursion, 90 observations for EPB excursion, and 210 observations were made for the displacement of the first dorsal compartment. Table 1 shows the mean excursion of the APL and EPB tendons before, after release and after Z-plasty of the first dorsal compartment of the wrist. A 2.5-mm (%29) increase in the excursion of the APL was observed after release (P < 0.05) while 0.8 mm (%.04) increase occurred after Z-plasty (P < 0.05). The EPB excursion increased by 2.18 mm (%30) after release (P < 0.05) and 0.54 mm (%0.07) after Z-plasty (P < 0.05). In comparison with various degrees of wrist flexion and extension, the most excursion of APL tendon was detected in neutral position (Fig. 2) and the most excursion of EPB tendon after release occurred at 0° and 30°dorsal flexion (Fig. 4). Table 2 demonstrates the mean displacement of the tendons after release of the compartment comparing the positions of the tendons before, after release and after Z-plasty. The mean displacement was 12.2 mm after release (p < 0.05) and 4.6 mm Z-plasty of the compartment (P < 0.05). The most displacement occurred with radial deviation while the least displacement was observed in ulnar deviation. The only exception occurs in 60° of dorsal flexion, where the radius allows ulnar deviation of the tendons. Fig. 5 shows the amount of displacement at various wrist positions. Our measurements show that the most displacement occurs at 30° of both palmar and dorsiflexion of the wrist. In palmar flexion, displacement occurred volarly, while in dorsiflexion, dorsal displacement was observed (Fig. 5).

**Table 1:** Mean of displacement (mm) of APL and EPB + SD.

Tendon	Excursion			
	Before release	After release	After Z-plasty	P value
**Abductor Pollicis** **Longus**	7.7 ± 1.62	9.9 ± 1.49	8.0 ± 1.49	P < 0.05
**Extensor pollicis** **Brevis**	7.3 ± 0.52	9.48 ± 0.90	7.84 ± 0.5	P < 0.05

**Table 2:** Displacement of APL and EPB tendons after release and after Z-plasty of the first dorsal compartment.

Wrist position	Radial		Neutral		Ulnar	
First compartment Condition	After release	After Z- plasty	After release	After Z- plasty	After release	After Z- plasty
Displacement of APL &EPB tendons (mm)	14.53	5.92	12.77	4.45	9.29	3.4
P value	<0.05	<0.05	<0.05	<0.05	<0.05	<0.05

## 4 Discussion

De Quervain's disease may lead to considerable disability and absence from work because of limited function of the hand and wrist. The assessment and treatment of de Quervain's tenosynovitis has developed since the first introduction by de Quervain in 1895. Up to now, conservative management such as local anesthetic and corticosteroid injections, strapping, cold, heat, rest, splints, massage and medications have been suggested [2,6,14,15]. When nonoperative treatment was not satisfactory after 4–6 months operative decompression of the first dorsal compartment might be considered [6,16]. Operative management of the first dorsal compartment for de Quervain tenosynovitis has been shown to be effective with a 91% cure rate. On the other hand, the possibility of surgical complications such as volar subluxation of the tendon, damage to the superficial branch of the radial nerve, and inadequate decompression should be considered [9,12,17]. Belsole et al. [4] evaluated 19 patients with de Quervain's disease after surgical decompression of the first dorsal compartment and found 36 complications including eight (22%) cases of volar tendon subluxation, eight (22%) patients with nerve injury, and seven (20%) cases of inadequate decompression. The rest of the complications (36%) were related to scars and incorrect diagnoses [18]. Additionally, some case reports of de Quervain's disease with symptomatic volar tendons subluxation were published [10,11,19]. Even though volar tendon subluxation is often asymptomatic, Volar subluxation of the tendon can lead to chronic tenosynovitis specially when the hand is used for heavy activities have need of wrist flexion and pinch [11,20,21]. We evaluated the results of the release and Z-plasty of the first dorsal compartment of the wrist that usually are done as a surgical treatment for de Quervain disease. There was not any difference in the increase of the excursion of the tendons at various degrees of the dorsal flexion and palmar flexion of the wrist. It may be due to the anatomic position of the first dorsal compartment that is nearly perpendicular to the wrist flexion extension arc of the motion. As might be expected, the bowstringing of the tendons were more in radial deviation and less in ulnar deviation. The only exception occurs in 60° of dorsal flexion, where the radius allows ulnar deviation of the tendons. In dorsal flexion there was more dorsal and in palmar flexion it was more volar displacement and bowstringing of the tendons. There are several reports about the effect of the flexor and extensor retinaculum of the wrist. Kline et al. [22] studied the effect of the release of transverse carpal ligament on the mechanics of the flexor tendons. They showed a 25% increase in the amount of excursion of the Flexor digitorum profundus and 20% for the flexor digitorum sublimis. They found 5.4±1 mm and 5.5 ± 1.3 mm bowstringing of each group of tendons respectively. Netscher et al. [23,24]in two separate studies evaluate the effect of division of the transverse carpal ligament on flexor excursion and in another study compared open and endoscopic CTR with repair of the transverse carpal ligament by use of proximally based flap. He found that flexor tendon excursion required achieving fingertip to palm contact to be uniformly increased for both FDS and FDP tendons when transverse carpal ligament was transected and that ratio of tendon excursion/digital flexion improved after transposition flap repair. Palmer et al. [25]on the anatomy and biomechanical study on fresh cadavers' specimen demonstrated that bowstringing of the extensor tendons occurred at complete resection of the dorsal retinaculum of the wrist. On the other hand they reported that not only some increase in the excursion of the tendons after release of the retinaculum was detected but also partial release of the extensor retinaculum prevents bowstringing and biomechanical changes of the extensor tendons. Our biomechanical study demonstrated that release of the first dorsal compartment of the wrist produces bowstringing and increase freedom of movement of the extensor tendons. This has clinical significance. Elftman suggested the relationship between fiber length, tension and excursion [26]. Brand studied the resting fiber lengths of the forearm muscles [27].By using the Elftman's length-tension relationship curve, and Brand's data for the resting fiber length of each extensor muscle, we calculated the decrease in the extensor and abductor powers. The measuring fiber length of the APL as calculated by Brand is 4.6 cm and for the EPB is 4.3 cm. Experimentally we found that release of the first dorsal compartment of the wrist resulted in increase of excursion of the APL tendon from 7.7 mm to 9.9 mm after release and from 7.7 to 8 after Z-plasty. These values for EPB are from 7.3 mm to 9.48 mm after release and from 7.3 mm to 7.84 mm after Z-plasty. Applying this to the adapted Elftman's curve, an increase of tendon excursion of 1 cm as a result of release of the first dorsal compartment would result in a decrease of tendon tension of approximately %65for APL and %80 for EPB. Given the fact that cadaveric tissues do not behave exactly as live tissues, this point should be considered as a limitation of the study. We concluded that Procedures such as enlargement plasty of Kapandji [9] or Z-plasty will increase the size of the compartment but will not change the biomechanical behaviors of the tendons significantly.

## Provenance and peer review

Not commissioned, externally peer-reviewed.

## Ethical approval

The ethical approval for the publication of this cadaveric case series was obtained by our institution (Louisville Arm and Hand surgery department).

## Sources of funding

This research did not receive any specific grant from funding agencies in the public, commercial, or not-for-profit sectors.

## Author contribution

1-Author name: Amit Gupta: Contribution (Type): Conception and design of the study. 2-Author name: Fateme Mirzaee: Contribution (Type): Drafting the article. 3- Author name: Amir Farahanchi Baradaran: Contribution (Type): Revising the article. 4- Author name: Mohammad Amin Aslani: Contribution (Type): Acquisition of data. 5- Author name: Hamidreza Aslani: Contribution (Type): Final approval of the version to be submitted & Corresponding author.

## Registration of research studies

Not applicable.

## Guarantor

Hamidreza Aslani, Shahid Beheshti University of Medical Sciences, Knee and Sport Medicine Research Center, Milad hospital, Tehran, Iran, phone: +98-21-88621147, +98–9121133968, Email: hraslani1342@gmail.com.

## Consent

Not applicable.

## Declaration of competing interest

The authors declare no conflicts of interest. The authors have no financial, consultative, institutional and other relationships that might lead to bias or conflict of interest.
